# Prospects of Marine Sterols against Pathobiology of Alzheimer’s Disease: Pharmacological Insights and Technological Advances

**DOI:** 10.3390/md19030167

**Published:** 2021-03-20

**Authors:** Md. Ataur Rahman, Raju Dash, Abdullah Al Mamun Sohag, Mahboob Alam, Hyewhon Rhim, Hunjoo Ha, Il Soo Moon, Md Jamal Uddin, Md. Abdul Hannan

**Affiliations:** 1 ABEx Bio-Research Center, East Azampur, Dhaka 1230, Bangladesh; ataur1981rahman@hotmail.com; 2 Center for Neuroscience, Brain Science Institute, Korea Institute of Science and Technology (KIST), Seoul 02792, Korea; hrhim@kist.re.kr; 3 Department of Anatomy, Dongguk University College of Medicine, Gyeongju 38066, Korea; rajudash.bgctub@gmail.com (R.D.); moonis@dongguk.ac.kr (I.S.M.); 4 Department of Biochemistry and Molecular Biology, Bangladesh Agricultural University, Mymensingh 2202, Bangladesh; sohag2010bmb.sust@gmail.com; 5 Division of Chemistry and Biotechnology, Dongguk University, Gyeongju 780-714, Korea; mahboobchem@gmail.com; 6 Graduate School of Pharmaceutical Sciences, College of Pharmacy, Ewha Womans University, Seoul 03760, Korea; hha@ewha.ac.kr

**Keywords:** cholesterol homeostasis, marine steroids, fucosterol, neurodegeneration, inflammation, oxidative stress

## Abstract

Alzheimer’s disease (AD) is a degenerative brain disorder characterized by a progressive decline in memory and cognition, mostly affecting the elderly. Numerous functional bioactives have been reported in marine organisms, and anti-Alzheimer’s agents derived from marine resources have gained attention as a promising approach to treat AD pathogenesis. Marine sterols have been investigated for several health benefits, including anti-cancer, anti-obesity, anti-diabetes, anti-aging, and anti-Alzheimer’s activities, owing to their anti-inflammatory and antioxidant properties. Marine sterols interact with various proteins and enzymes participating via diverse cellular systems such as apoptosis, the antioxidant defense system, immune response, and cholesterol homeostasis. Here, we briefly overview the potential of marine sterols against the pathology of AD and provide an insight into their pharmacological mechanisms. We also highlight technological advances that may lead to the potential application of marine sterols in the prevention and therapy of AD.

## 1. Introduction

Alzheimer’s disease (AD) is a devastating chronic neurodegenerative disorder characterized by intracellular aggregations of tau protein in neurofibrillary tangles (NFTs) formation and extracellular amyloid β-protein (Aβ) accumulation as the formation of a senile plaque in the specific brain regions [1,2]. About 70% of AD risk is found to be based on genetic predisposition, although numerous genes participate and its real causes in addition to molecular mechanisms have not been clearly elucidated [2,3,4]. However, aggregation of misfolded proteins could result in AD pathogenesis [5], and the extracellular domain along with a small cytosolic domain present in amyloid β-protein precursor (APP) is the key molecular driver of AD pathogenesis [6]. 

Despite the failure of recent clinical trials in antibody-based AD therapy [7], there is still hope for targeting AD-associated pathobiology by means of pharmacological agents. The therapeutic strategy of AD requires a multi-targeted approach because of its multifaceted pathobiology. Oxidative stress, neuroinflammation, and cholesterol dyshomeostasis constitute primary contributing factors in the pathogenesis of AD, and can, therefore, be potential targets for the development of anti-AD agents. Although synthetic and semi-synthetic drugs are the primary source of therapeutics against neurological diseases, including AD, their adverse side effects have led researchers to search for therapeutic leads in natural resources, such as the marine environment [8]. Approximately 70% of the Earth’s surface is covered by oceans, and diverse marine organisms offer a wonderful source of natural compounds [9]. Accordingly, recent observations have paid attention to the use of marine natural products that are relevant to treat AD [10]. Marine sterols, a class of sterol compounds, are such a group of natural molecules that are structurally and functionally similar to cholesterol, and their involvement in human health benefit and nutrition are imperative. Due to structural similarity and the sharing of the same absorption route, dietary sterols cause a reduction in intestinal cholesterol absorption and thereby play a significant role in maintaining cholesterol homeostasis, the disturbance of which is implicated in the pathobiology of various neurological diseases. 

Beyond their cholesterol-lowering potentials, marine sterols are shown to have therapeutic promise against AD by protecting against apoptosis, oxidative stress, and neuroinflammation through modulating cell survival pathways, such as brain-derived neurotrophic factor (BDNF), nuclear factor erythroid 2–2-related factor 2 (Nrf2), and nuclear factor kappa-light-chain-enhancer of activated B cells (NF-κB) signaling systems [11]. Despite the tremendous impact on neuropharmacology, much effort is required to achieve the use of marine sterols against AD in clinics. Here, we reviewed the neuropharmacological potentials of marine sterols against the pathobiology of AD and highlight technological advances towards the application of marine sterols in AD management.

## 2. Distribution and Pharmacokinetics of Marine Sterols

Marine sterols are distributed across several marine phyla (Table 1), and their pattern is influenced by geographic origin and ecological variation. Algae are among the marine organisms that contain an abundance of phytosterols, such as fucosterol, with significant pharmacological benefits [12]. Other marine organisms such as sponge [13], coral [14], and mollusk [15] differ in sterol contents; however, only a few of these sterols are important in neuropharmacology. 

Over the last few decades, pharmaceutical scientists have invested considerable interest in the modeling of in silico absorption, distribution, metabolism, excretion, and toxicity (ADME/T) as a rational drug design tool that plays an emerging role in drug development. The ADME/T profile of marine sterols was predicted using Schrodinger’s QikProp module, which provides ADME/T at a reliable level, describing drug likeliness and different pharmacokinetic parameters of compounds as shown in Table 1. Marine sterols were predicted to be potential drug-like molecules based on the comparison and range given at the bottom of Table 1. As reported here, fucosterol, the most abundant sterol of marine algae, conforms to Lipinski’s rule of five and Jorgensen’s rule of three, presenting its drug-likeliness. In addition, as the brain–blood partition coefficient (QPlogBB) of fucosterol is within the recommended range (−3.0–1.2), this sterol is likely able to cross the blood–brain barrier. Since marine sterols lack experimental data on pharmacokinetics, the in silico data that were incorporated in the review could provide future direction on studying pharmacokinetics and form a basis for the selection of a potential candidate in drug development. 

## 3. Pathobiology of Alzheimer’s Disease

Alzheimer’s disease (AD) is the most prevalent neurodegenerative disorder, contributing to dementia in the elderly. The amyloid plaque and neurofibrillary tangles (NFT) constitute the major pathological features of AD [33]. Oxidative stress and neuroinflammation are known to be among the primary causal factors in the pathobiology of AD [34,35]. When the generation of reactive oxygen species (ROS) exceeds the capacity of the cellular antioxidant defense system, a pathological condition called oxidative stress develops. Excitotoxicity, the exhaustive cellular antioxidant system, and brain susceptibility to lipid peroxidation contribute to OS [36]. ROS potentially causes damage by compromising the structure and function of cellular biomolecules that, in turn, cause neurodegeneration [37]. Neuroinflammation initiated by microglial activation culminates into chronic neurodegeneration [38]. Upon activation through toxicity, infection, and hypoxia, microglia secrete several pro-inflammatory and inflammatory cytokines [39] that stimulate neurons leading to neurodegeneration [40]. Imbalance in cholesterol homeostasis also may provoke OS and inflammation, thereby contributing to the pathobiology of AD [41]. Brain cholesterol metabolism is tightly regulated by the cholesterol transport mechanism. Upon activation, liver X receptor beta (LXR-β) upregulates multiple genes that encode proteins involved in the regulation of reverse cholesterol transport and thereby ensures neuroprotection [42,43]. For example, LXR-β agonist augmented amyloid β (Aβ) clearance [44]. Having association with pathobiology of AD, oxidative stress, inflammation, and cholesterol dyshomeostasis can be potential targets for therapeutic development.

## 4. Effects of Marine Sterols against Pathobiology of AD

Marine sterols, including fucosterol and saringasterol, were shown to be promising against AD by targeting oxidative stress, inflammation, cholinergic deficit, amyloidogenesis, cholesterol homeostatic pathway, and signaling systems that are linked with neuronal survival (Table 2). 

### 4.1. Protection against Oxidative Stress

**Figure 1:**
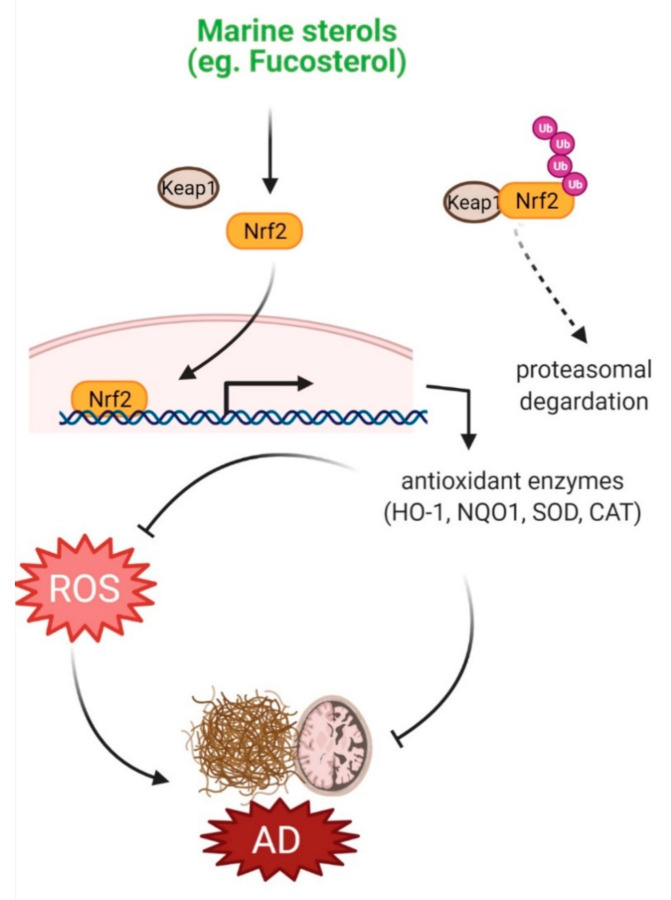
Effects of marine sterols on oxidative stress. Various sterols including fucosterol have been reported to activate Nrf2 signaling, which upregulates expression of various antioxidant enzymes, such as HO-1, NQO1, SOD and CAT. These enzymes inhibit ROS production and thereby may attenuate oxidative stress in AD pathology.

Fighting off oxidative stress, cells are equipped with antioxidant defense systems, comprising antioxidant enzymes such as catalase (CAT), glutathione peroxidase (GPx), and superoxide dismutase (SOD), and non-enzymatic antioxidants, such as glutathione and ascorbate. Dietary consumption of natural compounds can also strengthen the cellular antioxidant defense system through their adaptogenic potential [45]. Natural compounds can also target signaling pathways, including Nrf2/heme oxygenase-1 (HO-1), and thereby, potentiate intrinsic defense system [46]. Marine sterols were shown to protect against oxidative injury in various experimental models through their antioxidant property. Fucosterol and two other sterols, 3,6,17-trihydroxy-stigmasta-4,7,24(28)-triene and 14,15,18,20-diepoxyturbinarin, isolated from *Pelvetia siliquosa* protected against carbon tetrachloride (CCl_4_)-induced oxidative stress by enhancing SOD, CAT, and GPx1 levels in CCl_4_-challenged rats [20]. Fucosterol isolated from *Eisenia bicyclis* inhibited ROS production in tert-butyl hydroperoxide (t-BHP)-induced RAW264.7 macrophages [21]. In tert-BHP- and tacrine-challenged HepG2cell, fucosterol treatment caused a reduction in ROS and thereby attenuated oxidative stress by increasing glutathione level [22]. Fucosterol from *Sargassum binderi* protected against oxidative stress in particulate matter-induced injury and inflammation model of A549 human lung epithelial cells by accumulating SOD, CAT, and HO-1 in the cytosol, and Nrf2 levels in the nucleus [23]. A steroidal antioxidant, 7-dehydroerectasteroid F, isolated from the soft coral *Dendronephthya gigantea* was shown to protect against H_2_O_2_-induced oxidative damage in PC12 cells by enhancing nuclear translocation of Nrf2 and subsequent activation of HO-1 expression [16]. These protective effects of marine sterols against oxidative injury suggest their potential efficacy against oxidative stress-associated neurological disorders, including AD (Figure 1).

### 4.2. Protection against Neuroinflammation

In microglia challenged with extrinsic and intrinsic toxic stimuli, there is an elevated expression of inducible nitric oxide synthase (iNOS) and cyclooxygenase (COX-2), and secretion of inflammatory mediators such as tumor necrosis factor-α (TNF-α), interleukin-6 (IL-6), and interleukin-1β (IL-1β), which can stimulate neurons to cause degeneration, ultimately leading to AD. Natural products, including phytosterols that attenuate inflammatory signals can be beneficial in the management of AD [53,54,55]. Mounting evidence suggests anti-inflammatory potentials of marine sterols. Fucosterol treatment of lipopolysaccharide (LPS)- or Aβ-stimulated microglial cells ameliorated inflammation by lowering the secretion of IL-1β, IL-6, TNF-α, nitric oxide (NO), and PGE2 [24]. Fucosterol attenuated the inflammatory response in LPS-stimulated RAW 264.7 macrophages by downregulating COX-2 and iNOS expression and suppressing NF-κB signaling [21]. Fucosterol can also attenuate LPS-mediated inflammation by suppressing NF-κB activation and stimulating alveolar macrophages [56]. In CoCl_2_-challenged cells, fucosterol inhibited inflammatory response by lowering the production of TNF-α, IL-6, and IL-1β [26]. Fucosterol attenuated particulate matter-induced inflammation by inhibiting activation and nuclear translocation of NF-κB and phosphorylation of p38 mitogen-activated protein kinase (MAPK), extracellular signal-regulated kinases 1/2 (ERK1/2), c-Jun N-terminal kinases (JNK), and COX-2 [23]. Fucosterol of *Undaria pinnatifida* downregulated the transcription of iNOS, TNF-α, and IL-6, and inhibited their production. Moreover, fucosterol inhibited LPS-mediated activation and nuclear translocation of NF-κB. In addition, fucosterol attenuated activation of mitogen-activated protein kinase kinases 3/6 (MKK3/6) and MAPK-activated protein kinase 2 (MK2) of the MAPK pathway, suggesting that the anti-inflammatory effects of fucosterol may be, at least in part, associated with the inactivation of NF-κB and p38 MAPK pathways [25].

**Figure 2:**
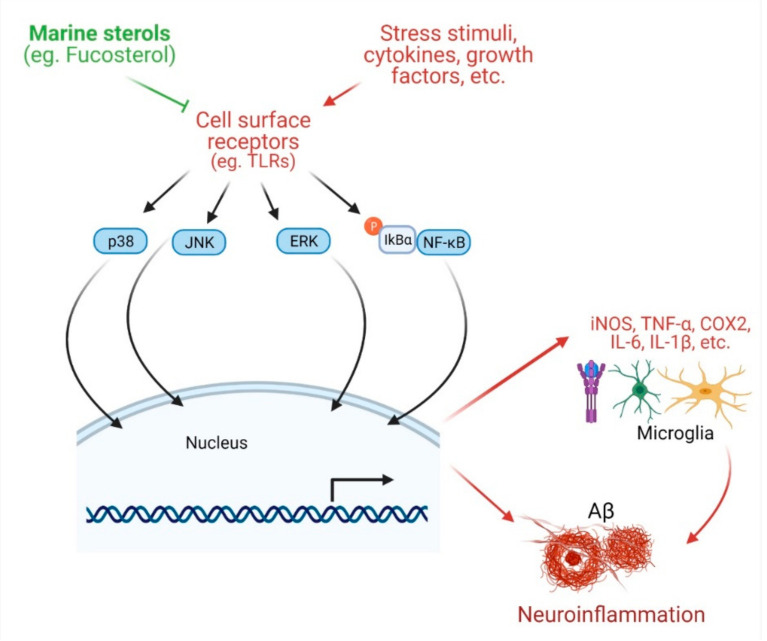
Effects of marine sterols on inflammation. Various stress stimuli, growth factors, and cytokines bind with diversified cell surface receptors (such as TLRs) and mediate different downstream signaling pathways, such as p38 MAPK, JNK, ERK, and NF-κB. These enter into the nucleus for transcription of various pro-inflammatory cytokines, including iNOS, TNFα, COX2, IL-6, and IL1β. All of these ultimately help in the formation of Aβ plaque in brain. Various sterols including fucosterol have been reported to disturb the cell surface receptors as well as major signaling systems leading to inhibition of inflammatory response.

Apart from algal sterols, there are some other marine sterols that are also important as anti-inflammatory agents. Two steroids, 5α-pregn-20-en-3β-ol and 5α-cholestan-3,6-dione, isolated from an octocoral *Dendronephthya mucronate*, were shown to inhibit LPS-induced NO production in activated RAW264.7 murine macrophage cells [18]. Another octocoral sterol, dendronesterones D, isolated from *Dendronephthya* sp., inhibited the expression of iNOS and COX-2, and thereby protected against inflammation [17]. Anti-inflammatory effects of marine sterols suggest their potential in protecting against neuroinflammation in AD pathology (Figure 2).

### 4.3. Marine Sterols as Cholinesterase Inhibitors

The cholinergic deficit has been established as a clinical consequence of AD pathology. Cholinesterase inhibitors that can temporarily slow down cholinergic neurotransmission can improve AD outcomes. Marine sterols have also been shown to inhibit the activity of cholinesterase. Fucosterol and 24-hydroperoxy 24-vinylcholesterol showed inhibition against butyrylcholinesterase (BChE) with IC_50_ values of 421.72 ± 1.43 and 176.46 ± 2.51 μM, respectively [27]. In another study, fucosterol exhibited dose-dependent inhibition against acetylcholinesterase (AChE) and BChE activities [24]. Enzyme kinetics and structural analysis demonstrated that fucosterol acts as a non-competitive inhibitor to AChE [47].

### 4.4. Marine Sterols as β-Secretase Inhibitors

**Figure 3:**
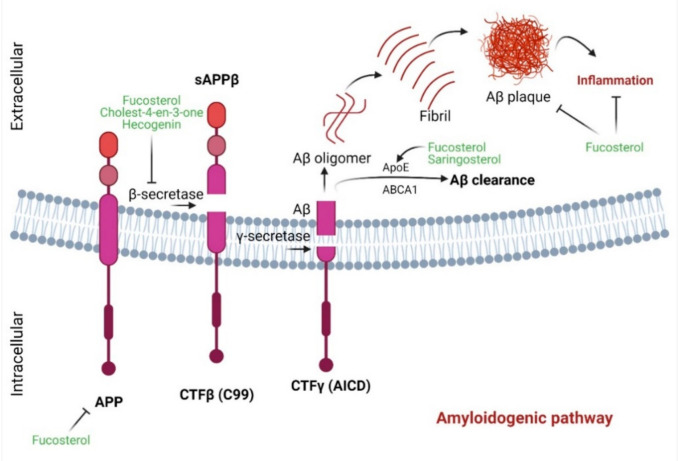
Effects of marine sterols on APP processing pathways in AD. In the amyloidogenic pathway, APP is cleaved by β-secretase, which produces a soluble amyloid precursor protein β (sAPP β) and a C-terminal fragment β (CTFβ) or C99 fragment. The C99 fragment is cleaved by γ-secretase to generate Aβ and C-terminal fragment γ (CTFγ) or AICD. Further, Aβ constructs Aβ oligomers which ultimately form fibrils and Aβ plaques. Interestingly, fucosterol and other marine sterols inhibit β-secretase, protect against Aβ-mediated inflammation and promote Aβ-clearance.

The aggregation of Aβ represents a characteristic hallmark of AD. β-secretase, which catalyzes the initial breakdown of amyloid precursor protein (APP) to generate Aβ, may represent a promising target for the development of an anti-AD agent [57]. However, evidence suggests that complete inhibition of β-secretase activity might have unintended sequelae with behavioral deficits [58]. Natural products that bear reversible and non-competitive binding patterns with β-secretase may therefore bear therapeutic promise against AD. Natural products, including marine sterols, possess anti-amyloidogenic potential. Fucosterol can be such a potential candidate due to its anti-β-secretase activity [48]. The mode of inhibition is of noncompetitive type, indicating that fucosterol could be an effective and safer inhibitor. Additionally, as shown in computational analysis, fucosterol can be docked on the active site of β-secretase via hydrogen bonding and hydrophobic interactions [59]. Moreover, fucosterol shows competitive binding energies of −10.1 [48] and −19.88 kcal/mol [59], respectively, indicating that hydrogen bonding may ensure close association with enzyme active site, leading to a more effective β-secretase inhibition. Moreover, hecogenin and cholest-4-*en*-3-one isolated from fat innkeeper worm *Urechis unicinctus* exhibited anti-β-secretase activity with EC50 of 390.6 µM and 116.3 µM, respectively [29]. With this evidence, these marine sterols can be a potent anti-amyloidogenic agent for use against AD (Figure 3).

### 4.5. Marine Sterols as Neuroprotective Agent

**Figure 4:**
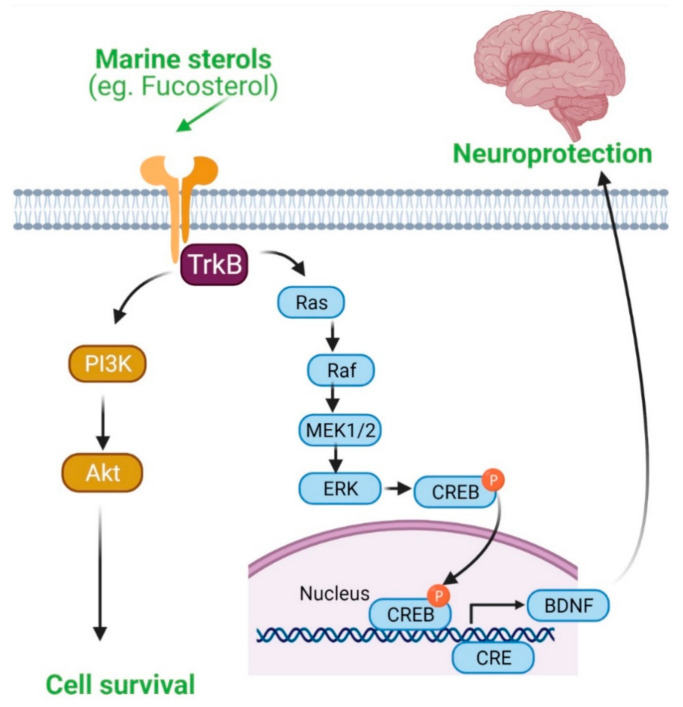
Activation of BDNF-dependent pro-survival pathway by fucosterol. TrkB/PI3K/Akt and TrkB/ERK signaling pathways are involved in neuroprotection.

Aβ aggregation initiates neuroinflammation and thereby can contribute to the pathobiology of AD. Marine sterols have been shown to protect against Aβ-induced cytotoxicity and clear Aβ in several studies. Fucosterol protected against Aβ_1–42_ (sAβ_1–42_)-mediated cytotoxicity and suppressed glucose-regulated protein 78 (GRP78) expression in cultured hippocampal neurons by upregulating tropomyosin receptor kinase B (TrkB)-mediated ERK1/2 signaling [49] (Figure 4). These in vitro effects of fucosterol were further translated into an in vivo model, in which fucosterol co-treatment ameliorated sAβ_1–42_-induced cognitive impairment in aging rats through suppression of GRP78 expression and upregulation of BDNF expression in the dentate gyrus [49]. In Aβ-induced SH-SY5Y cells, fucosterol pretreatment attenuated neurotoxicity by upregulating neuroglobin (Ngb) mRNA expression [50]. Fucosterol preconditioning also decreased APP mRNA and lowered Aβ levels in activated SH-SY5Y cells [50]. Supplementation of astrocytes with 24(S)-saringosterol caused an increase in ApoE secretion. Furthermore, supplementation of microglia with conditioned medium of 24(S)-saringosterol-treated astrocytes augmented microglial clearance of Aβ_1–42_. 24(S)-saringosterol reduces Aβ_42_ release in APP overexpressing neuronal N2a cells [30]. 16-*O*-desmethylasporyergosterol-β-d-mannoside isolated from marine-derived fungus *Dichotomomyces cejpii* exhibited a moderate Aβ-42 lowering activity in APP-overexpressing aftin-5-treated N2a cells [28]. 4-methylenecholestane-3β,5α,6β,19-tetraol attenuated glutamate-induced neuronal injury, prevented N-methyl-D-aspartate (NMDA)-induced intracellular calcium increase, and inhibited NMDA currents, suggesting that this marine-derived sterol could also have therapeutic potential against glutamate excitotoxicity [51].

### 4.6. Marine Sterols as Regulators of Cholesterol Homeostasis

Cholesterol is known to regulate cell-to-cell communication and transmembrane signaling [60], and is critical in the development and maintenance of central nervous system (CNS) neurons. A defect in cholesterol metabolism results in synaptic dysfunction, oxidative stress and inflammation, triggering the onset of AD pathology [61]. Activation of LXR-β upregulates several genes of reverse cholesterol transport, including apolipoprotein E (ApoE), ATP-binding cassette transporter (ABCA1), ATP binding cassette subfamily G member 1 (ABCG1), and sterol regulatory element-binding protein 1 (SREBP1), and thereby this nuclear receptor plays a significant role in the protection against neurodegeneration [42,43]. Upon ligand activation, LXR-β attenuated dopaminergic loss [62] and reduced the toxic burden of mutant huntingtin [63], and also accelerated Aβ clearance [44]. Experimentally, acting as a selective LXR-β agonist, fucosterol augmented the expression of LXR target genes encoding ABCA1, ABCG1, and ApoE [31,52]. This evidence demonstrates that fucosterol may produce similar LXR-β-mediated effects to aid in brain cholesterol homeostasis and play a pivotal role against AD pathology involving Aβ clearance via ABC/SHREBP1/ApoE-dependent pathways (Figure 3). Saringasterol is also a selective LXRβ agonist and promoted the transcriptional activation of ABCA1, ABCG1, and SREBP-1c in multiple cell lines and thus is suggested to be a potent natural cholesterol-lowering agent [31].

## 5. Pharmacological Mechanism of Protective Actions of Marine Sterols against AD Pathology

Marine sterols confer neuroprotection by attenuating various factors implicated in the pathobiology of AD, including oxidative stress, inflammation, Aβ_1−42_-induced apoptosis, and cholesterol dyshomeostasis. Antioxidant activity of marine sterols has been manifested by their capacity to promote expression of enzymatic (such as SOD, GPx, CAT, and HO-1) and non-enzymatic (such as GSH) antioxidants, and normalize various oxidative markers (such as ROS; malondialdehyde, MDA; lipid hydroperoxide, LPO and 4-Hydroxynonenal, 4-HNE) (Figure 1). As activation of Nrf2 results in the upregulation of over 250 genes that encode proteins of antioxidant defense systems [64], overexpression of this transcription factor in marine sterols-treated cultures [16,23] indicates the involvement of the Nrf2 signaling system. 

Another potential mechanism of sterol-mediated neuroprotection involves anti-inflammation, which is indicated by their capacity to inhibit the release of proinflammatory and inflammatory mediators (such as IL-1β, IL-6, TNF-α, NO, and PGE2) and the expression of inflammatory enzymes (such as NOS, and COX2) and to downregulate the activation and subsequent nuclear translocation of transcription factor NF-κB, and phosphorylation of MAPK, ERK1/2 and JNK [17,21,23,24] (Figure 2). Yet, another potential mechanism is that the reverse cholesterol transport system under the influence of marine sterols that induces expression of LXR target genes such as ABCA1, ABCG1, and ApoE regulates cholesterol homeostasis in the brain and can prevent AD progression by playing an important role in Aβ clearance (Figure 3). Furthermore, the cell survival system, such as the TrkB-mediated ERK1/2 signaling pathway, is implicated in sterol-mediated antiapoptotic effects in Aβ-induced hippocampal neurons (Figure 4). In addition, BDNF expression by sterol treatment also plays a crucial role in ameliorating memory impairment in Aβ-induced aging rats (Figure 4).

## 6. Technological Advances toward Sterol Therapy

After the discovery of cholesterol-lowering potentiality, dietary sterols have taken their place in the global market as nutraceuticals supplements, available either in tablet or capsule forms [65]. When administrated, sterols integrate into the mixed micelles in the intestinal chyme and compete with cholesterol to be transported to the enterocyte. Once transported, sterols, however, elated back out from enterocytes into the lumen with the help of ABCG5/G8 system [66]. The ABCG5/G8 system is also responsible for the excretion of sterols that are available in the circulatory system and chylomicrons via the liver biliary system [67]. Therefore, an optimal delivery system or formulation of sterols is necessary to enhance subsequent pharmacological activities. 

Sterols are slightly soluble in oil, insoluble in water, and can exist as a crystalline powder. To increase the water solubility, phytosterol esterification was first introduced and used in the first commercial functional food product, margarine [68]. Esterification allows phytosterol to be dissolved in the oil to a ten-fold greater degree than usual and also shows no effect in food texture and test. It was postulated that smaller particle size sterols are more soluble in water than the large size one [69]. However, Keller et al. [70] found no difference in tissue distribution between the customary and nanoscale size of free phytosterol in the hamsters, and also no significant decrease in total cholesterol level was observed. In addition, several methods to date have been adopted to enhance the solubility of sterols, by incorporating free sterols into functional foods and center around reducing crystallization. As an example, Leong et al. constructed sterol nanodispersions by using the emulsification-evaporation technique in the various organic solvents, where they found that larger phytosterol nanoparticles can be produced through a higher organic: aqueous phase ratio and higher homogenization pressure. Furthermore, hexane allowed for obtaining the smallest particle size [71]. Likewise, several methods such as supersaturation using crystallization inhibitors [72], emulsion with lecithin [73], the rapid expansion of supercritical solution into an aqueous solution [74], and microemulsion by solvent displacement [75] are beingly considered. Ling and Lin showed that the bioavailability of sterols can be improved by using the microencapsulation method using in vitro release analysis [76]. In the respective study, they used oven-dried kenaf seed oil containing microencapsulated sterols, where chitosan and alginate with high methoxy pectin were used as shell materials. Ubeyitogullari et al. developed a novel approach to produce low crystallinity phytosterol nanoparticles, which improved both bioaccessibility and bioavailability of phytosterol. In the study, phytosterol nanoparticles were formulated by nanoporous starch aerogels, in combination with supercritical carbon dioxide, wheat starch, and corn starch aerogels. This combination improves sterols’ bioavailability by 20 fold when impregnated into wheat starch aerogels monolith [77]. Meng et al. proposed a method to enhance the stability and bioavailability of sterols by formulating hydroxypropyl β-cyclodextrin sterols inclusion complex. Their study showed that the inclusion complex enhanced water solubility of sterols to 8.68 mg mL^−1^ and resulted in free form 0.02 mg mL^−1^ [78]. Likewise, many studies have recently been conducted to enhance the bioavailability of sterols, but no studies have focused on brain delivery [79,80,81,82]. Sterol-loaded nanocarriers seem promising to increase more bioavailability in blood; however, more extensive studies are required to investigate tissue and organ distributions and the toxicity risks. 

## 7. Concluding Remarks and Future Perspectives

This review highlights the neuroprotective potential of marine sterols against AD pathobiology and provides an insight into the underlying molecular mechanisms. Substantial evidence shows that marine sterols protect against AD-associated pathological factors such as apoptosis, oxidative stress, and neuroinflammation by adapting cell survival pathways, such as BDNF, Nrf2, and NF-κB signaling systems and attenuate cholesterol imbalance by activating LXR-mediated reverse cholesterol transport mechanism, and thereby can prevent, or at least slow down, AD progression, suggesting that these marine natural products can be potential candidates in the development of anti-AD agents. 

Despite significant progress, marine sterols, such as common phytosterols, are still far from clinical applications. Additional investigations are highly recommended to further elucidate the exact mechanisms of action of marine sterols. Since the existing evidence on the neuroprotective efficacy is based on preclinical studies, human clinical trials with appropriate study protocols are crucial to further characterize the beneficial roles of marine sterols as well as to recommend for future clinical use against AD.

The possible advantages of considering marine sterols in clinical application stand by their multitargeted actions in the pathobiology of AD. Moreover, marine sterols share common features and functionality of cholesterol and other biological sterols, in particular, stigmasterol and β-sitosterol, which have shown promise in clinical trials against various chronic diseases [83]. With technological advances, including microencapsulation or nanoparticle-based drug delivery, marine sterols may offer potential lead chemicals in developing viable anti-AD therapeutics.

## Figures and Tables

**Table 1:** Distribution and ADME/T properties of marine sterols with known neuroactive roles.

Sterol	Distribution	Structure	ADME/T Properties									
			Lipinski’s Rule of Five				Jorgensen’s Rule of Three			Blood–Brain Barrier Permeability		Percent Human Oral Absorption
			mol_MW	donorHB	accptHB	QPlogPo/w	QPlogS	QPPCaco	#metabolites	QPlogBB	CNS	
7-dehydroerectasteroid-F	Soft coral *Dendronephthya gigantea* [16]	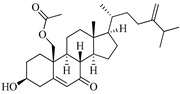	470.691	1	5.7	5.766	−7.264	494.218	5	−1.288	−2	95.962
Dendronesterones-D	Octocoral *Dendronephthya* sp. [17]	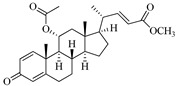	440.578	0	6	4.652	−6.582	308.75	2	−1.253	−2	100
5α-cholestan-3,6-dione	Octocoral *Dendronephthya mucronate* [18]	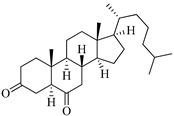	400.643	0	4	5.731	−7.143	1210.653	4	−0.683	0	100
Fucosterol	Brown algae [19,20,21,22,23,24,25,26]	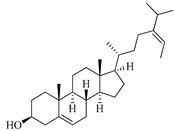	412.698	1	1.7	7.577	−8.812	3376.384	6	−0.299	0	100
24-hydroperoxy-24-vinylcholesterol	*E. stolonifera* [27]	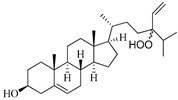	444.696	2	4.15	6.183	−7.195	1183.894	3	−0.947	−1	100
16-*O*-desmethylasporyergosterol-β-d-mannoside	Fungus *Dichotomomyces cejpii* [28]	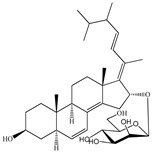	572.781	5	11.9	3.639	−6.171	149.465	11	−2.149	−2	74.215
5α-pregn-20-en-3β-ol	Octocoral *Dendronephthya mucronate* [18]	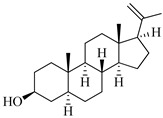	316.526	1	1.7	5.097	−5.957	3378.51	3	0.019	1	100
Cholest-4-en-3-one	Fatworm *Urechis unicinctus* [29]	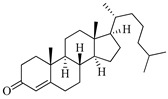	384.644	0	2	6.923	−8.177	2769.384	2	−0.316	0	100
Saringosterol	Brwon algae [30,31]	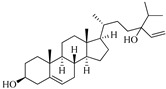	428.697	2	2.45	6.912	−7.854	1981.099	4	−0.655	0	100
24-methylenecholestane-3β,5α,6β,19-tetraol	Soft coral *Nephthea brassica* [32]	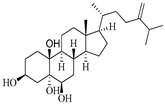	434.658	4	4.9	5.105	−6.979	665.416	6	−1.315	−2	94.407

mol_MW: Molecular weight of the molecule. (130.0–725.0); donorHB: estimated number of hydrogen bonds that would be donated by the solute to water molecules in an aqueous solution. (0.0–6.0); accptHB: estimated number of hydrogen bonds that would be accepted by the solute from water molecules in an aqueous solution. (2.0–20.0); QPlogPo/w: predicted octanol–water partition coefficient. (−2.0–6.5); QPlogS: predicted aqueous solubility, log S. (−6.5–0.5); QPPCaco: predicted apparent Caco-2 cell permeability in nm/sec. (500: great); #metabolites: number of likely metabolic reactions. (1–8); QPlogBB: predicted brain–blood partition coefficient. (−3.0–1.2); CNS: predicted central nervous system activity on a −2 (inactive) to +2 (active) scale. (−2 (inactive), +2 (active)); Percent Human Oral Absorption: predicted human oral absorption on 0 to 100% scale. (>80% is high); Rule of Five: number of violations of Lipinski’s rule of five [3]. The rules are: mol_MW < 500, QPlogPo/w < 5, donorHB ≤ 5, accptHB ≤ 10. Compounds that satisfy these rules are considered druglike. (maximum is 4); Rule of Three: number of violations of Jorgensen’s rule of three. The three rules are: QPlogS > −5.7, QP PCaco > 22 nm/s, # Primary Metabolites < 7. Compounds with fewer (and preferably no) violations of these rules are more likely to be orally available. (maximum is 3).

**Table 2:** Comprehensive summary on protective effects of marine sterols against Alzheimer’s disease (AD) pathology.

Anti-AD Effects	Name of Sterol	Marine Source	Dose Regimen	Experimental Model	Major Findings	Reference
Protection against oxidative stress	Fucosterol, 3,6,17-trihydroxy-stigmasta-4,7,24(28)-triene and 14,15,18,20-diepoxyturbinarin	*Pelvetia siliquosa*	30 mg/kg/day for 7 days prior to CCl_4_ challenge	CCl_4_-stimualted Rat model	↑SOD, CAT, and GPx	[20]
	Fucosterol	Edible brown alga *Eisenia bicyclis*	25–400 μM	tert-BHP-induced RAW 264.7 macrophage cells	↓ROS generation	[21]
		*Ecklonia stolonifera and Eisenia bicyclis*	25–100 μM	tert-BHP- and tacrine-induced HepG2cell injury model	↓ROS generation ↑GSH level	[22]
		Brown alga *Sargassum Binderi*	3.125–100 μg mL^−1^	CPM-stimulated injury and inflammation in A549 epithelial cells	↓ROS level ↑SOD, CAT, and HO-1 in cytosol, and Nrf2 in nucleus	[23]
	7-dehydroerectasteroid F	Soft coral *Dendronephthya gigantea*	10 μM	H_2_O_2_-induced oxidative damage in PC12 cells	Nuclear translocation of Nrf2 and ↑HO-1	[16]
Protection against inflammation	Fucosterol	*Panida australis*	0.004, 0.2, and 10 μM	LPS- and Aβ-induced BV2 (microglial) cells	Attenuates LPS- or Aβ-induced inflammation ↓IL-6, IL-1β, TNF-α, NO, and PGE2	[24]
		*Eisenia bicyclis*	5–20 μM	LPS-stimulated RAW 264.7 murine macrophages	↓NO production ↓iNOS and COX-2 ↓NF-κB pathway	[21]
		Brown seaweed *Undaria pinnatifida*	10, 25, or 50 μM	LPS-induced RAW 264.7 macrophage and THP-1 human monocyte cell line	↓iNOS, TNF-α, and IL-6 ↓DNA binding ↓phosphorylation of NF-κB, MKK3/6 and MK2	[25]
		*Hizikia fusiformis*	1–10 μM	CoCl_2_-induced hypoxia in keratinocytes	↓IL-6, IL-1β and TNF-α ↓pPI3K and pAkt and HIF1-α accumulation	[26]
		*Sargassum binderi*	3.125, 6.25, 12.5, 25, 50, 100 μg mL^−1^	CPM-stimulated injury and inflammation in A549 epithelial cells	↓COX-2, PGE2, TNF-α and IL-6 ↓nuclear translocation of NF-κB and phosphorylation of MAPK, ERK1/2 and JNK	[23]
	5α-pregn-20-en-3β-ol and 5α-cholestan-3,6-dione	Octocoral *Dendronephthya mucronate* (Cnidaria)	IC50 of 30.15 ± 1.05 and 35.97 ± 2.06 μM, respectively	LPS-induced RAW264.7 murine macrophage cells	↓NO formation	[18]
	Dendronesterones D	Octocoral *Dendronephthya* sp.	10 μM	LPS-induced RAW264.7 macrophage cells	↓iNOS and COX-2	[17]
Anticholinesterase activity	Fucosterol and 24-hydroperoxy 24-vinylcholesterol	*E. stolonifera*	IC_50_ values of 421.72 ± 1.43, 176.46 ± 2.51 µM, respectively	Enzymatic assay	Selective inhibition of BChE	[27]
	Fucosterol	*Panida australis*	Anti-AChE (10.99–20.71%) and anti-BChE (4.53–17.53%) activities with concentrations ≤ 56 μM	Enzymatic assay	Nonselective cholinesterase inhibition	[24]
		*Sargassum horridum*	-	In vitro enzymatic assay	↓AChE activity (Non-competitive inhibition)	[47]
β-Secretase inhibitory activity	Fucosterol	*Eckloniastolonifera* and *Undaria pinnatifida*	10-100 μM (IC_50_ 64.12 ± 1.0 μM)	In vitro enzymatic assay and In silico analysis	↓β-secretase activity (Noncompetitive inhibition)	[48]
	Cholest-4-*en*-3-oneand hecogenin	*Urechis unicinctus*(fat innkeeper worm or marine spoon worm or penis fish)	EC50 390.6 µM and 116.3 µM, respectively	Fluorescence Resonance Energy Transfer (FRET)-based enzyme assay	Anti-BACE1 activity was comparable to curcuminoids, terpenoids, and tannins	[29]
Neuroprotectiveactivity	Fucosterol	*Ecklonia stolonifera*	1–10 µM at 24 h before sAβ_1-42_ challenge (effective fucosterol conc. 5–10 µM)	sAβ_1–42_ (10 µM) -induced ER stress model of primary neurons and sAβ_1–42_-induced memory dysfunction in aging rats	Reduces apoptosis in Aβ_1–42_-stimulated cytotoxicity and ameliorates Aβ_1–42_-induced cognitive decline ↑TrkB-mediated ERK1/2 signaling ↓GRP78 expression ↑BDNF expression	[49]
		*-*	0.0032 to 20 μM	Aβ-stimulated cytotoxicity in SH-SY5Y cells	Attenuates apoptosis in Aβ-induced SH-SY5Y cells ↑Ngb mRNA ↓APP mRNA and Aβ levels	[50]
	24(S)-Saringosterol	*Sargassum fusiforme*	10 µM	Microglia-treated conditioned medium of 24(S)-Saringosterol-treated astrocytes; Mouse neuroblastoma (N2a)-APP695 cells	Aβ_1−42_ clearance; ↓Aβ-42 secretion; LXRβ activation	[30]
	16-*O*-desmethylasporyergosterol-β-d-mannoside	Fungus *Dichotomomyces cejpii*	10 μM	Aftin-5 treated N2a-APP695 cells	Moderate Aβ-42 lowering activity	[28]
	24-methylenecholestane-3β,5α,6β,19-tetraol	Soft coral *Nephthea brassica*	10 μM	Glutamate-induced neuronal injury	Promote cell survival; Negative modulation of NMDA receptor	[51]
Cholesterol homeostasis	Fucosterol	-	100 or 200 μM	HEK293 cell cultures (Reporter system); THP-1-derived macrophages, Caco-2 cells and HepG2 cells	Reverses cholesterol transport; Nonselective LXR agonist ↑ABCA1, ABCG1, and ApoE ↑Intestinal NPC1L1 and ABCA1 ↑Insig-2a, that delays nuclear translocation of SREBP-1c	[52]
	Saringosterol	*Sargassum fusiforme*	30 μM	Luciferase reporter assay system; HEK293T, THP-1 monocytes, HepG2, RAW264.7, THP-1 macrophages and Caco-2 cells	Selective LXRβ agonist. ↑ABCA1, ABCG1, and SREBP-1c	[31]
